# Differing views regarding diet and physical activity: adolescents versus parents’ perspectives

**DOI:** 10.1186/s12887-020-02038-4

**Published:** 2020-03-27

**Authors:** Kristen M. J. Azar, Meghan Halley, Nan Lv, Sharon Wulfovich, Katie Gillespie, Lily Liang, Lisa Goldman Rosas

**Affiliations:** 1 Sutter Health Center for Health Systems Research, 795 El Camino Real, Ames Building, Palo Alto, CA 94301 USA; 2 Stanford University, Stanford, USA; 3 University of Illinois, Chicago, USA; 4 UC San Diego School of Medicine, San Diego, USA

**Keywords:** Adolescent health, Lifestyle intervention, Childhood obesity

## Abstract

**Background:**

Today, approximately one in five United States adolescents age 12 to 19 years is obese and just over a third are either overweight or obese. This study examines how parents and peers influence diet and physical activity behaviors of older adolescents (14–18 years) with overweight or obesity to inform weight management interventions.

**Methods:**

Adolescent participants included 14 to 18-year-olds with a Body Mass Index (BMI) greater than the 85th percentile for their age and sex who were receiving care in a large healthcare system in Northern California. Adolescents and their parents participated in separate focus groups and interviews (if not able to attend focus groups) that were held at the same time in the same location. We used qualitative thematic analysis to identify common themes discussed in the adolescent and parent focus groups as well as paired analysis of adolescent-parent dyads.

**Results:**

Participants included 26 adolescents and 27 parents. Adolescent participants were 14 to 18 years old. Half were female and the participants were almost evenly distributed across year in school. The majority self-identified as White (56%) and Asian (36%).Three themes were identified which included 1) parents overestimated how supportive they were compared to adolescents’ perception 2) parents and adolescents had different views regarding parental influence on adolescent diet and physical activity behaviors 3) parents and adolescents held similar views on peers’ influential role on lifestyle behaviors.

**Conclusion:**

Parents’ and adolescents’ differing views suggest that alignment of parent and adolescent expectations and behaviors for supporting effective weight management could be incorporated into interventions.

## Background

The prevalence of obesity among adolescents age 12 to 19 years in the United States (US) has doubled over the past two decades. Today, approximately one in five US adolescents age 12 to 19 years is obese and just over a third are either overweight or obese [1]. Obesity in adolescence is associated with numerous immediate and long-term adverse health [2–6] and psychosocial consequences [3, 5–7]. For example, the prevalence of prediabetes/diabetes is estimated to be 23% among US adolescents [8]. Additionally compared to normal weight peers, obese adolescents are more likely to suffer from depression [9], shame [10], bullying [11], and anxiety [12] – factors that may contribute to weight gain and hinder engagement in interventions. Furthermore, obesity during adolescence is associated with an increased risk of obesity in adulthood [13]. According to national data, 90% of obese adolescents remained obese over a decade later, suggesting a high likelihood that obesity in adolescence persists into adulthood [14]. Obesity in adulthood is associated with costly and debilitating conditions including diabetes, cardiovascular disease, and some cancers [15]. Thus, supporting adolescents to achieve a healthy weight will improve their immediate health and well-being as well as decrease their risk of obesity and its related comorbidities in adulthood.

Despite the urgent public health need for treating obesity in adolescence, there is a paucity of evidence-based interventions, especially for older adolescents age 14–18. Adolescence is a distinct development period in which children transition to adulthood and increasingly gain autonomy [16], including in lifestyle behaviors such as diet and physical activity [17–21], providing a crucial window of opportunity for establishing sustainable, lifelong healthy habits [18]. This developmental period is also marked by increasing dependence on peer relationships with a continued importance of parents and the family unit as a central influence on adolescents’ lifestyle. These factors underscore the need for tailored approaches to address this unique developmental period.

In younger children direct involvement of parents is a key factor for success [22–24]. While obesity tends to run in families [25–27] and parents remain an important influence for adolescents [28], the importance of peers and increasing autonomy make it difficult to know how best to engage parents in adolescent weight management programs. To date, research findings have been mixed regarding the extent to which involving parents is beneficial for adolescent weight loss [29].Al-Khudairy et al. found that behavioral interventions for adolescents that involved parents, compared to those with no parental involvement, did not seem to differ in effectiveness for weight-related outcomes [30]. Limited research offers differing views on the impact of parental involvement in adolescents. Engagement with adolescents and their parents to explore their views is important for informing successful weight management interventions for this age group.

To fill a critical gap in our understanding regarding successful weight management strategies for this age group, we engaged adolescents and their parents using separate focus groups to examine how parents and peers influence diet and physical activity behaviors of older adolescents (14–18 years) with overweight and obesity. The ultimate goal of this study was to directly inform the development of a primary care-based weight management intervention for adolescents age 14 to 18 with a BMI > the 85th percentile for age and sex.

## Methods

### Sample and recruitment

Participants included 14 to 18-year-old adolescents with a Body Mass Index (BMI) greater than the 85th percentile for their age and sex who were receiving care within a large multispecialty healthcare system in Northern California. The Sutter Health Institutional Review Board approved all study procedures (PAMF# 14–03-302EXP). Electronic Health Records (EHR) were used to identify potentially eligible current patients based on age and BMI percentile. We identified individuals with an encounter or clinic visit including a measurement of weight and height in the past 6 months and used this to calculate BMI for eligibility criteria. Exclusion criteria included serious physical or mental health conditions listed in the EHR and per physician discretion. Physicians involved in the study were consulted regarding participant eligibility and were asked to review the list of potential participants from their patient panel and indicated any patients they viewed as inappropriate for the study (based on serious physical or mental health impairment) as well as referred additional patients to be recruited. There were no specific inclusion or exclusion criteria applied to parent participants. Once approval was obtained, 377 invitation letters were mailed to parents/guardians [referred to as “parents” in the remaining text] from their adolescent’s pediatrician or family medicine doctor explaining the study and inviting their adolescent to participate with an option to opt out of the study. Parents who did not opt out through phone or reply card received a call from study staff to assess interest in participation and screen for eligibility. After parents consented for their adolescent to participate, they were invited to participate in a separate parent focus group. All participating parents provided written informed consent and adolescents provided written assent.

### Data collection

Study staff trained in qualitative methods facilitated three focus groups with adolescents and three with their parents in summer 2015. For participants who were not able to attend the focus groups, trained study staff conducted individual in-depth interviews with adolescents (*n* = 5) and with their parents (*n* = 4). Adolescents and their parents participated in separate focus groups and interviews that were held at the same time in the same building. The focus group/interview guides for both adolescents and parents covered five main topic areas: 1) causes and consequences of overweight, 2) experiences or attempts with diet and weight loss, 3) diet, 4) physical activity, and 5) family and peer influences. Questions were open-ended and attempted to foster discussion. Probing questions were asked when necessary to deepen the discussion. All focus groups and interviews were audio recorded and transcribed verbatim.

Participant demographics were assessed using a self-administered survey prior to the focus group discussion. In addition, participants completed a survey that included questions about lifestyle activities and decision-making. The parent version assessed parents’ opinions for themselves and their adolescent for each topic. Adolescents received $20 for participating in a 2-h focus group or a 1-h interview; the participating parents did not receive any monetary incentive or compensation.

### Data analysis

We summarized findings from the survey using descriptive statistics. For the focus groups transcripts, we used an inductive approach including two phases for the qualitative analysis given the broad topics covered in the focus groups and interviews. In the first phase, two co-authors (KG and LL) came to consensus on an initial set of codes and their definitions based on reviewing the first 2 focus group and 2 interview transcripts. Through discussion among all study team members, the codebook was refined and finalized. KG and LL conducted the coding of all the focus group and interview transcripts according to the codebook using Dedoose (Version 7.0.23), a web-based software for qualitative and mixed-methods analysis. All respondents were assigned a study identifier with a linking identifier for adolescent-parent dyads, which was applied as a code to corresponding excerpts throughout all transcripts. This method allowed adolescents’ data that were coded with a linking identifier to match responses from their parent in order to facilitate comparisons between adolescent and parent responses within the same family.

The data were then reviewed by code to identify common themes both within and across the focus groups/interviews, with data summarized for adolescents and parents separately for comparison to identify overarching themes for each group. In addition, paired parent-adolescent responses were examined by code to identify similarities and discrepancies in accounts of adolescents’ diet and physical activity routines and their attempts to improve their diet or increase physical activity. KG and LL summarized coding reports and created an overall summary of key themes for parents and for teens separately, and of key areas of inconsistency between parent and teen reporting. Not all teen-parent dyads had a quote for every theme. Adolescent and parents’ demographic and lifestyle surveys were quantitatively summarized using means and proportions using SPSS (Version 14.0).

## Results

### Participant characteristics

A total of 26 adolescents and 27 parents participated in the focus groups and interviews. Adolescent participants ranged in age from 14 to 18 years old (Table 1). Half of the group was female and the participants were almost evenly distributed across year in school. Of the adolescent participants, the majority self-identified as White (56%) and Asian (36%). Parent participants were mostly female (81%), married (85%), had completed either college or graduate school (72%) and more than half reported annual household income greater than $150,000 (64%) (Table 1). Slightly more than a third (37%) were born outside of the US. This sample is similar to the population served by the health care system. The underlying catchment area from which the study participants were recruited spans five counties, has a higher average household income of $106,489 [31] and a relatively large Asian population (29%) [32]. Given this, the representativeness of this sample to the broader U.S. population is limited.

**Table 1:** Adolescent and parent sociodemographic survey results

Characteristic	Adolescent (***n*** = 26)	Parent^**a**^ (***n*** = 27)
**Age, mean (SD)**	15.5 (1.2)	48.2 (6.0)
**Sex, n (%)**		
Female	13 (50)	22 (81)
Male	13 (50)	5 (18)
**Married or living with partner, n (%)**		
Yes	–	23 (85)
No	–	4 (15)
**Insurance status, n (%)**^**b**^		
Private	–	24 (89)
Public	–	2 (6)
Other	–	1 (3)
**Parental education level, n (%)**		
Some college or less	–	7 (28)
College graduate	–	11 (44)
Graduate school	–	7 (28)
**Parental Annual Income, n (%)**		
$25,001 to $50,000	–	1 (3)
$75,001 to $100,000	–	2 (7)
$100,001 to $150,000	–	5 (19)
$150,001 to $200,000	–	5 (19)
$200,001 or higher	–	9 (33)
Don’t know/prefer not to answer	–	4 (15)
**Adolescent grade level, n (%)**		
9th Grade	6 (23)	–
10th Grade	6 (23)	–
11th Grade	7 (26)	–
12th Grade	6 (23)	–
Other	1 (3)	–
**Race/ethnicity**^**c**^		
Hispanic/Latino	4 (15)	4 (15)
American Indian	1 (4)	–
Asian	9 (36)	9 (33)
Black or African American	2 (8)	2 (7)
White	14 (56)	16 (59)
Other	2 (8)	–
**Parent country of birth, n (%)**		
US	–	17 (63)
Outside of US	–	10 (37)

^a^One parent signed the consent form and completed a survey but did not participate in the focus group

^b^More than one selection could be made for Health Insurance

^c^More than one selection could be made for race/ethnicity

### Emergent themes and findings

Three themes emerged from the focus groups and interviews with adolescents and parents. Survey findings are included throughout to support qualitative findings.

#### Theme 1: Parents and adolescents differ in their perception of parental support for weight management

In general, the focus groups/interviews and survey indicated that parents and adolescents differed in their perceptions of adolescents’ weight management needs, although there were a few examples of agreement. Paired analysis of adolescents and parents from their respective focus group transcripts revealed that one-third (*n* = 9) of parents reported aiding their adolescent in their change attempts for healthy diet; but only 15% (*n* = 4) of adolescents reported receiving aid from their parent(s) in their change attempts. There were also substantial discrepancies between adolescents’ accounts of their parents’ assistance and parents’ own accounts of their assistance. Among one parent-adolescent pair, the parent reported helping her adolescent with portion control and planning meals (Table 4, quote 1.1) while the adolescent complained that her mother offered little support in providing healthier food (Table 4, quote 1.2) and expressed a general lack of positive support from her mother (Table 4, quote 1.3). Among another parent-adolescent pair, the parent reported that despite her efforts to introduce whole grains, increase vegetables and salads, and replace unhealthy snacks (e.g. cookies) with healthier ones (e.g. fruit), the adolescent was reluctant to eat healthier foods. In contrast, the adolescent reported having tried to eat less “chips and sweets” but said it was very difficult due to lack of healthy food options at home.

From the survey data it was apparent that parents and adolescents differed in their report of prior weight loss attempts (Table 2). Almost 70% of adolescents reported having tried to lose weight while only about half of the parents (48%) reported that their adolescent had done so. More than half of the adolescents indicated that they had tried to increase their physical activity (62%) and decease their sugar intake from candy and sweets (62%), less than one-third of parents shared this view (23 and 31% respectively). In contrast, the majority of parents (69%) indicated that their child had attempted to lose weight by reducing “junk food or fast food” intake, while less than half (47%) of adolescents agreed. Further, 78% of the adolescents reported eating more fruits, vegetables and salads as a weight loss method, only 46% of the parents indicated that their adolescent had been doing so.

**Table 2:** Adolescents’ weight loss attempts

Question: How did you (your adolescent) try to lose weight? (check all that apply)	Adolescent (***N*** = 18) N (%)	Parent about Adolescent (***N*** = 13) N (%)
Ate more fruits, vegetables, and salads	14 (78)	6 (46)
Increased moderate physical activity, such as walking	11 (62)	3 (23)
Ate less sugar, candy, or sweets	11 (62)	4 (31)
Switched to foods with lower calories	10 (55)	6 (46)
Ate less junk food or fast food	10 (55)	9 (69)
Switched to foods with fewer carbohydrates (carbs)	8 (44)	6 (46)
Started an exercise regimen, such as running or going to the gym	7 (32)	6 (46)
Switched to foods with less fat	5 (28)	4 (31)
Joined a weight loss program such as Weight Watchers	0	0
No answer	0	1 (7)

There were some examples of both the adolescent and parent agreeing that the parents had provided assistance to help adolescents eat healthier. For instance, one adolescent reported that he had started eating healthier foods and his parents had been helping him in the process (Table 4, quote 1.4). The parent also shared more details about how he had helped the adolescent eat healthier by helping with portion control (especially with pasta), providing a lot of healthy options at home and taking the adolescent to grocery store with him (Table 4, quote 1.5). In addition, this adolescent and parent pair both reported doing a 7-min workout as a family every Saturday for almost a year.

#### Theme 2: Parents and adolescents had different views regarding parental influence on adolescent diet and physical activity behaviors

The focus groups/interviews revealed that adolescents desired support from their parents while parents reported that adolescents sought out peer support for eating healthy. For example, adolescents liked the fact that parents helped them to eat healthy meals (Table 4, quote 2.1). Adolescents also reported that their parents served as role models for healthy eating. One adolescent noted that her mother’s weight loss efforts made it easier for her to change her own diet (Table 4, quote 2.2). Another adolescent shared a similar experience, even though she sometimes found the emphasis on healthy food “annoying” (Table 4, quote 2.3). In response to one mother’s recounting of her efforts to encourage her adolescent to be more physically active, another parent expressed his opinion that parent influence becomes less important and their peer group becomes more important at this age. (Table 4, quote 2.4).

The differing views of parental influence on diet were also apparent when both groups were asked about perceived control over what the adolescents eat on the survey (Table 3). Among the adolescents, about half (54%) expressed a feeling of complete control and an additional 35% expressed some control over what they eat at home, while parents responded that the control was shared, with 67% saying their child had “some control” over what is eaten. At school, 53% of adolescents felt they had complete control over their diet while only 41% of the parents felt this was true of their teens. In focus groups/interviews, parents acknowledged their role in supporting healthy eating by discussing their efforts to provide healthy meals for dinner but almost all concurred that they have little to no control over adolescents’ lunch. Some parents expressed that adolescents exerted their own will in planning lunch and two parents reported that their adolescents once mentioned that bringing lunch to school is "not cool".

**Table 3:** Adolescents’ and Parents’ Perception of Teen Control Over Diet

	Complete control		Some control		Little control		No control	
	Teen	Parent	Teen	Parent	Teen	Parent	Teen	Parent
**At home, n(%)**	14 (54)	8 (30)	10 (36)	18 (67)	0	1 (4)	2 (8)	0
**At school, n(%)**	14 (54)	11 (41)	11 (42)	12 (44)	1 (4)	3 (11)	0	0

In regard to physical activity, focus groups and interviews revealed that some adolescents wanted support for physical activity from parents and some did not while parents unanimously thought that their support was not influential. Some adolescents reported that they enjoy working out with their parents and found them great motivators (Table 4, quote 2.5). On the other hand, some adolescents complained that they disliked working out with their parents for various reasons. For example, one adolescent was not happy with her mom not sticking to exercising together and complaining too much (Table 4, quote 2.6). Another adolescent described his experience with working out with parents as embarrassing, at least initially (Table 4, quote 2.7). Meanwhile, parents felt strongly that adolescents need support from their peers more than support from parents to become more physically active. For example, a parent noted that support from peers rather than nagging from parents is better because peers go through the same issues together (Table 4, quote 2.8). Another parent reported great frustration over "nagging" her adolescent in vain and that she had already given up on trying (Table 4, quote 2.9).

**Table 4:** Quotes organized by theme and order of appearance

	Quote	Said by
**Theme 1: Parents and adolescents differ in their perception of parental support for weight management**		
1.1	*"We just talked about eating smaller meals and making more of a plan of when you’re going to eat. And kind of looking at what you can eat* versus *what, you know - you still get pleasure and that satisfaction with something else. And then we reduced the size, and we made it six small meals."*	43 year old Parent
1.2	*“Adolescent: No, and she kept buying awful foods, and she just kept eating them in front of me. And I was like, ‘Could you not do that?’ Like, I’m trying to focus on this, and you’re just not making it any easier.”*	15-year-old female
1.3	*“I mean, it’s been pretty easy, except when my mom makes a dinner that doesn’t have, like, anything healthy. Like, we’re going to have macaroni and hot dogs for dinner. It’s like, oh, okay. Are there any, like, greens involved or any fruit or anything? Just like, no, just this. I was like, oh, okay.”*	15-year-old female
1.4	*“I started maybe two months ago, to eat healthier......choose the low-sugar version of granola cereals … my parents had helped me to try to eat healthier … and I’ve been trying to-I’ve definitely been cutting down on pasta and stuff.”*	14-year-old male
1.5	*“So, if he were to have a really big bowl of pasta or something like that I might say, ‘Eh, that’s a lot of pasta. You might want to –‘So, sometimes he’ll say, ‘Okay, you’re right,’ and he’ll put some of the pasta back … What we try to do to make it easier for him is we try to have a lot of options that he likes. So, instead of saying eat your whatever it is, we’ve slowly added a lot of fruits and vegetables that he likes … … He likes things like - he’ll have bell peppers and he likes apples and carrots. Cauliflower he likes. Pineapple, cantaloupe. I try to always - when I go to the store, get things that I know that he likes and then, occasionally, I’ll try to take him so that he has a choice of picking out things that he’s interested in trying.”*	52-year- old parent
**Theme 2: Parents underestimate their influence on adolescent lifestyle behaviors (e.g. diet and physical activity)**		
2.1	*“I’m pretty balanced. My parents go get, like, equal parts meat and vegetables, fruit, and so I don’t have to worry about that.”*	17-year-old male
2.2	*“Well my mom has been dieting a lot lately. She actually just lost like 50 pounds ever since our dad left a year ago. I guess that if other people that you are spending time with are making an effort then it’s easier for you to make an effort too because you are with them and that kind of thing.”*	15-year-old female
2.3	*“My mom’s, like, super pro-healthy, so she’s always just going out of her way to make sure, like, dinner’s good for us. It’s kind of annoying sometimes … … But, like, yeah, dinner’s really there’s a huge variety in what we eat, so it’s pretty good.”*	16-year-old female
2.4	*[Female 1, 43] “My husband and I, we do yoga every morning, and then we walk. And, you know, ‘Come on, [name], let’s go.’ And every now and then, she’ll do it. But I’m not going to drag her, you know? It has to be that internal motivation thing. But so, yeah, she does dance at school, and so …* *[Male, 56] At this age group, too, I think we become less important in that, and their peer group becomes more important.* *[Female 2, 43] Yeah, absolutely.* *[Female 1, 43] Yes.*	Multiple
2.5	*“I actually enjoy working out with my dad, because he kind of motivates me, because he does triathlons and half marathons. So, he kind of set the goal for me and tried getting me into it. So, whenever I practice with him or just go biking, he’s usually ahead of me and makes me catch up with him and just work harder. And so, I feel like that’s good for me.”*	15-year-old female
2.6	*"Honestly I hate working out with my mom, because we tried this one thing together, and she’s like, “Oh, we both need to work out together,” and I was like, “Okay.” So, we started doing it, and then she just kept complaining, and then she quit, and then I just kept doing it. And then she’s like, “Oh, I want to join you again.” And I was like, “Okay.” Then she kept complaining, and then she quit. And it’s just a constant thing. Like, she just can’t stick with it. I’m kind of like, “Okay, either you want to do it or you don’t.”*	15-year-old female
2.7	*“I’m fine with working out with my parents. It’s just, like, a little embarrassing at first. You don’t want them to see you being, like, different from them, so then it’s a little embarrassing. But after that, you get used to it, and it’s fine.”*	17-year-old male
2.8	*"I think it’ll be better for him … … .there’s other kids who are like him and they can all - his peer group and go through the same types of issues together. I think for (my adolescent) that’s better than the adults always telling him. I think he’s tired of the adults saying, ‘Okay, you need to eat better. You need to do this, you need to do that"*	58-year-old parent
2.9	*“I can’t physically put him someplace with his own initiative. So I kind of gave up pestering him because I feel like I’m always criticizing him and always nagging him about all these things, the room, the food. I feel like almost every time I address him, it’s going to be in a negative way. That makes me feel really bad, but I’m trying to help him.”*	53-year-old parent
**Theme 3: Parents and adolescents held different views regarding peer influences on lifestyle behaviors (e.g. diet and physical activity)**		
3.1	*“Like one of my friends lives like literally 3 houses away from this big plaza where they have a McDonald’s, Taco Bell, 7-Eleven, like all these places and she just goes there all the time and when she doesn’t go there she has like ramen noodles. She never has any home cooked meals. She is like the worst one of my friends but she is like really skinny and she just has one of those metabolism things and she brags about it all the time. It’s super annoying.”*	15-year-old female
3.2	*“When I’m in control of them, yeah, they’re healthy. But if he’s with his friends, then ColdStone is the meal, or something like that.”*	56-year-old parent
3.3	*“Being with my friends and stuff, like doing something that we enjoy doing. A lot of my friends are into sports and basketball … …*”	15-year-old male
3.4	*“It’s like different friends I guess … … the one that didn’t leave is the one that sits around all day. But those people [friends who have just moved away] would kind of kick my butt sometimes and say like hey lets go for a hike. And I went on a hike with them twice.”*	17-year-old male
3.5	*“I mean there were a couple of times where his friends stopped by and said, ‘We’re going to the gym to go work out’, so he would walk with them. I think that if his friends did that more or maybe if we did it as a family more and not as busy - but I think if his friends did it more I think he would be more apt to go because I don’t think he wants to hang out with his family at the gym*.”	47-year old parent

#### Theme 3: Parents and adolescents held similar views regarding peer influences on lifestyle behaviors (e.g. diet and physical activity)

Adolescents and parents generally agreed that peers negatively influenced diet. Adolescents generally described the influence of their peers on diet as negative because they tended to consume low-cost or junk food and sugar-sweetened beverages when they were spending time with their peers. For some adolescents, observations of their peers’ behavior, as opposed to any direct peer pressure to eat unhealthy foods, was a source of frustration and cause for discouragement. For example, one adolescent noted that one of her friends always boasts about not gaining weight from eating junk food frequently, which frustrated her (Table 4, quote 3.1). Parents agreed with adolescents and tended to describe peer influence on diet as negative and resulting in unhealthy food choices. For example, a parent described how she was not able to control what her adolescent ate when he was with his friends (Table 4, quote 3.2).

With regard to physical activity, adolescents and parents reported that the effect of peers varied. Adolescents described that peers can have both a negative and positive influence on physical activity. Some adolescents described feeling motivated to engage in planned physical activities with friends such as a hike or other types of group exercise (Table 4, quote 3.3). However, others reported negative influence by friends such as feeling uncomfortable working out with friends who are in better shape. Other adolescents commented that their peers rarely do any exercise, implying a social norm among that peer group. Another adolescent described how different friends influenced his physical activity (Table 4, quote 3.4), contrasting a group of friends who encouraged him to hike with them with another friend who preferred to be sedentary. Parents agreed with adolescents that peers negatively and positively influenced adolescents’ physical activity. Peers negatively influenced physical activity if they were not interested in physical activity (e.g., engage in screen time when they spend time together). Peers positively influenced physical activity if they were interested in being active when they spend time together. For example, one parent thought that her adolescent would be more willing to do exercise with friends versus with family (Table 4, quote 3.5).

## Discussion

In this study we found that adolescents and parents held different views on the role that parents can play in weight management and in the perceived influence of parents versus peer influence on health behaviors like diet and physical activity. In general, adolescents viewed their parents as having an important influence on weight management, healthy eating, and physical activity. Parents felt that their role was less important than that of peers for influencing their adolescents’ diet and physical activity behaviors. We also found that adolescents and parents agreed that peers negatively influence diet and have mixed effects on physical activity, based on the activity level of the peers. The information on adolescent and parent perceptions can be used to inform weight management interventions that are developmentally appropriate for this age group.

The results of this study argue for the inclusion of parents in interventions for older adolescents, contrasting some prior studies that argue for the lack of significant impact [33] or disadvantage to parental involvement [34]. There is a large amount of evidence that supports the positive impact of parental involvement in weight management interventions for younger pediatric populations [22, 23, 35, 36], but less is known about how to optimize parental involvement for older adolescents. These approaches will need to balance this age group’s increasing autonomy and reliance on peers with their continued need for parental support. Possible approaches include increasing parents’ ability to serve as a role model for weight management at home [35], and providing parents with skills for effective communication strategies for weight management with adolescents [37]. Additionally, the focus groups and interviews inform suggestions for how parents might support the specific health behaviors of healthy eating and physical activity.

For diet, adolescents highlighted the important role that parents play in creating a healthy food environment in the home. It is estimated that adolescents consume 63–65% of their daily calories at home [38]. The paired analysis revealed that increased communication around what the home food environment should include is needed. Availability of healthy foods like vegetables, fruits and balanced meals was as important as an absence of junk foods and sugar-sweetened beverages. These strategies could target the parents directly as well as indirectly through supporting adolescents to have conversations with their parents about the home food environment. Goal setting with parents and adolescents around the home food environment may be an effective strategy. For physical activity, some adolescents were open to physical activity with their parents while the parents unanimously reported that peers as opposed to parents were the most important. Additionally, while some adolescents reported that their parents were positive role models for physical activity, the fact that their parents were not physically active did not seem to deter adolescents from engaging in physical activity. Observational research has shown that parents who encourage and value physical activity consequently influence children’s behaviors, resulting in higher levels of physical activity among them [39–41]. A relatively recent meta-analysis [35] found that support from parents and their modelling behaviors were related to adolescent physical activity. Strategies that help adolescents to identify successful social support mechanisms may reveal individualized approaches to physical activity promotion. Strategies for parents may be to not assume that their adolescent children do not want to participate in physical activity with them, but rather to have conversations about the role they may play.

Adolescents and parents generally agreed that peers have a negative influence on diet, primarily due to the foods that they eat when they spend time with their friends. This is in line with other studies that have found that adolescents tend to consume unhealthy foods when they spend time together and that peers may encourage the consumption of unhealthy foods within social groups [20, 42, 43]. However, there is limited empirical evidence to elucidate how peer factors may be related to adolescents’ unhealthy food intake and some studies support the importance of bolstering self-regulation [44]. Strategies informed by this finding on peer influence may include equipping adolescents with skills to maintain healthy eating when they spend time with friends. For example, adolescents may benefit from problem solving skills that would help them to successfully navigate situations when they want to eat healthy and they are with peers. Additionally, skills that support adolescents in effectively communicating why they are making healthy choices may help them to maintain a healthy diet. Finally, fostering positive peer social support through bringing together adolescents who are seeking to make dietary changes may be effective. Problem solving, effective communication skills, and social support are all hallmark strategies in evidence-based weight management programs such as the Diabetes Prevention Program that are designed for adults [45]. These programs may be able to be adapted for older adolescents.

In contrast to the finding on diet where peers were largely a negative influence, adolescents and parents agreed that peers can have both a positive and negative influence on physical activity. They acknowledged that adolescents want to engage in activities with their peers. Thus, if the peers are engaged in physical activity, this will have a positive effect and if they are engaged in sedentary activities this will have a negative effect [46–49]. The implications of the effect of peers on physical activity has several important implications for weight management strategies. Encouraging adolescents to identify and plan for activities that they can engage in with their friends has potential for increasing physical activity. Recognizing that peer support for physical activity is important, interventions could also create opportunities for adolescents to be physically active together.

### Limitations

This study analyzed the views and experiences of 26 adolescents and 27 parents from Northern California and participants were recruited from one healthcare system. Given the scope of this study, we were not able to analyze adolescents who were overweight separately from those who were obese. Future work should aim to elucidate potential differences and commonalities in the experiences, lifestyle behaviors and perspectives for these distinct therapeutic groups. While we included adolescent participants with a BMI that is over the 85th percentile for age, we did not capture the weight of parent participants. There is a possibility that parent support for or assistance with weight management maybe related to their own weight. Due to this limitation, it is unclear whether pattern of parent behavior in support/nonsupport of healthy food choices and/or physical activity may be related to parent weight status. This would be an interesting and important consideration for future research. Additionally, given the recognized importance of home environment and family influence on lifestyle behaviors it would be important for future studies to explore the influence of family size (i.e., siblings) on adolescent behaviors. Finally, patients of this healthcare system may be different from the general population. Future research, with a larger, representable sample of adolescents and parents would be helpful to verify the study’s results, including the comparison between views of adolescents and those of parents. Given that the prevalence of overweight/obesity are higher among individuals/families of low income/social advantage, compared with high income/social [50, 51] advantage. Repeating the study among adolescents/families who are socially disadvantaged would be an important priority for future work as well.

## Conlcusion

This study provides perspectives of both parents and their older adolescents on a pressing public health issue. The findings of this study affirm the need for interventions that aim at initiating and supporting effective communication between parents and older adolescents in order to promote effective weight management efforts for adolescents at risk for adulthood obesity. More work is needed to explore barriers and facilitators to effective communication as well as methods to overcome barriers and improve communication specifically regarding older adolescent weight management.

## Data Availability

The datasets generated and analyzed during this study are not publicly available due to institutional policies.

## Abbreviations

BMI: Body Mass Index
US: United States
EHR: Electronic Health Records
